# Mortality of Chicago

**Published:** 1846-02

**Authors:** Wm. B. Herrick

**Affiliations:** Professor of Anatomy in Rush Med. College


ILLINOIS

MEDICAL & SURGICAL JOURNAL.

VOL. II. FEBRUARY, 1846. NO. 11.

### MORTALITY OF CHICAGO.

*By* Wm. B. Herrick, *Prof. of Anatomy in Rush Med. College.*

As appears by a record, kept by A. S. Bates, City Sexton, the proportion of deaths to the population of Chicago, during the years 1843, '44, and '45, inclusive, were as follows:

| Years. | Deaths. | | Inhabitants. | | Death. | | Inhabitants. |
|---|---|---|---|---|---|---|---|
| In 1843 | 117 | to | 7,580 | making | 1 | to | 64.78 |
| " 1844 | 288 | " | 10,170 | " | 1 | " | 38.78 |
| " 1845 | 290 | " | 12,088 | " | 1 | " | 41.68 |

Making an average for the 3 years, viz: 1843, '44, and '45, of 1 in 48.

Comparing these statistics with those of Philadelphia, New York, London, and Paris, and taking an average of their mortality for ten years, from 1821 to 1830 inclusive, we find the proportions as follows:

| | | Death. | | Inhabitants. |
|---|---|---|---|---|
| | Philadelphia, | 1 | to | 38 |
| | New York, | 1 | " | 36 |
| | London, | 1 | " | 55 |
| | Paris, | 1 | " | 36 |
| From 1843 to '45 inclusive, | Chicago, | 1 | " | 48 |

It will be seen by this comparison that the average degree of mortality in Chicago is less than in either of the above named cities, London alone excepted. Thus it is proven beyond all controversy, contrary to the general opinion abroad, that this city is remarkable for the low proportion of its deaths, and of course is as healthy, in point of location, as any city in the country, Philadelphia not excepted.

In 1845, from January to October inclusive, there were in Chicago 239 deaths, as follows:

| | Whole No. Deaths. | Adults. | Children. |
|---|---|---|---|
| In January, | 19 | 5 | 14 |
| " February, | 18 | 6 | 12 |
| " March, | 11 | 8 | 3 |
| " April, | 15 | 10 | 5 |
| " May, | 22 | 16 | 6 |
| " June, | 9 | 6 | 3 |
| " July, | 42 | 16 | 26 |
| " August, | 35 | 16 | 19 |
| " September, | 48 | 15 | 33 |
| " October, | 20 | 11 | 9 |
| Total, | 239 | 109 | 130 |

Referring again to the statistics of Philadelphia and New York, we find that in those cities, during the same months in 1830, the proportions were as follows:

| | Deaths. | Adults. | Children. |
|---|---|---|---|
| In Philadelphia | 3,435 | 1,769 | 1,666 |
| " New York, | 4,665 | 2,056 | 2,609 |
| Same months, 1845, Chicago, | 239 | 109 | 130 |

By the above comparison it appears that the proportion of deaths among children, compared with the mortality at all ages, is greater in Chicago than in Philadelphia, but less than in New York, even when no allowance is made for the comparatively large proportion of children constituting our population. When in comparing statistics, due allowance is made for this peculiarity,—the unusual number of children in the population of our city,—it is found that the mortality among them is less even than in most other places. Hence the accusation against Chicago. that children here are remarkably subject to disease, finds no foundation in fact.

The above tables also show, that the greatest mortality both among adults and children is during the months of July, August, and September, caused, without doubt, by the prevalence, during the hot weather of this period, of Miasmatic and abdominal diseases, such as remittent, intermittent, bilious, and congestive fevers, inflammations of the stomach, intestines and liver, diarrhœa, dysentery, &c., all of which diseases are every where more prevalent during the summer months.

The next greatest mortality is in the months of January and February, a period in which inflammatory diseases of the air passages and lungs, are most common and severe.

The above named diseases causing doubtless two-thirds of the deaths in our city, are not often fatal when met by early, judicious and prompt treatment, but it is a fault too common with our citizens, to call a physician too late, or, what is worse, to seek the advice of a practitioner, in whose peculiar sugar and water treatment, there is neither harm nor efficiency.

The above statistics for three years show a proportion of deaths, as follows: 1 in 64 in 1843, 1 in 38 in 1844, and 1 in 41 in 1845, showing a great increase of mortality since 1843, which can only be accounted for as follows:

During the last named two years, epidemics, such as scarlet fever, &c., have been more prevalent, our citizens have become more active in manufacturing, building, and commerce; and of course more workmen are employed, liable to a greater extent than others to accidents. But above all, homœpathic practice has come into vogue since 1843, and is still blindly adhered to by many of our citizens, which by its inefficient treatment has *permitted*, as is the opinion of all our most intelligent physicians, at least one-third of the deaths from the above named most common causes.

